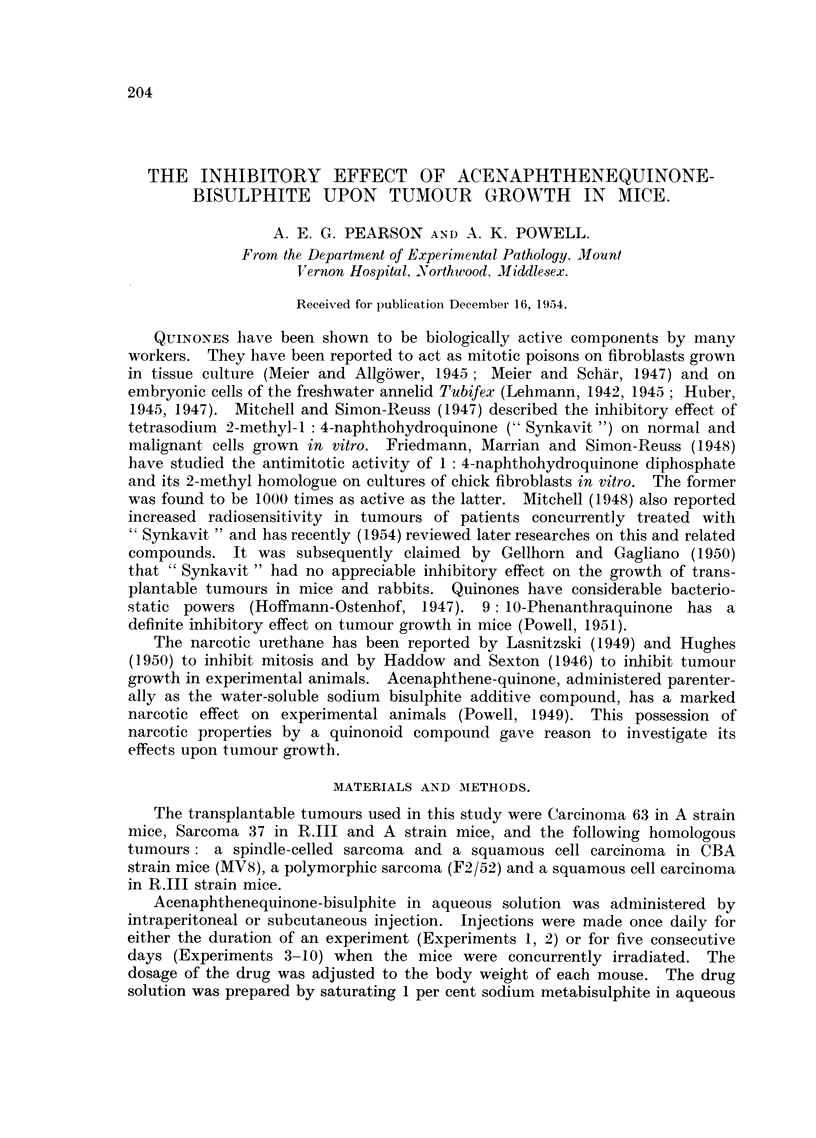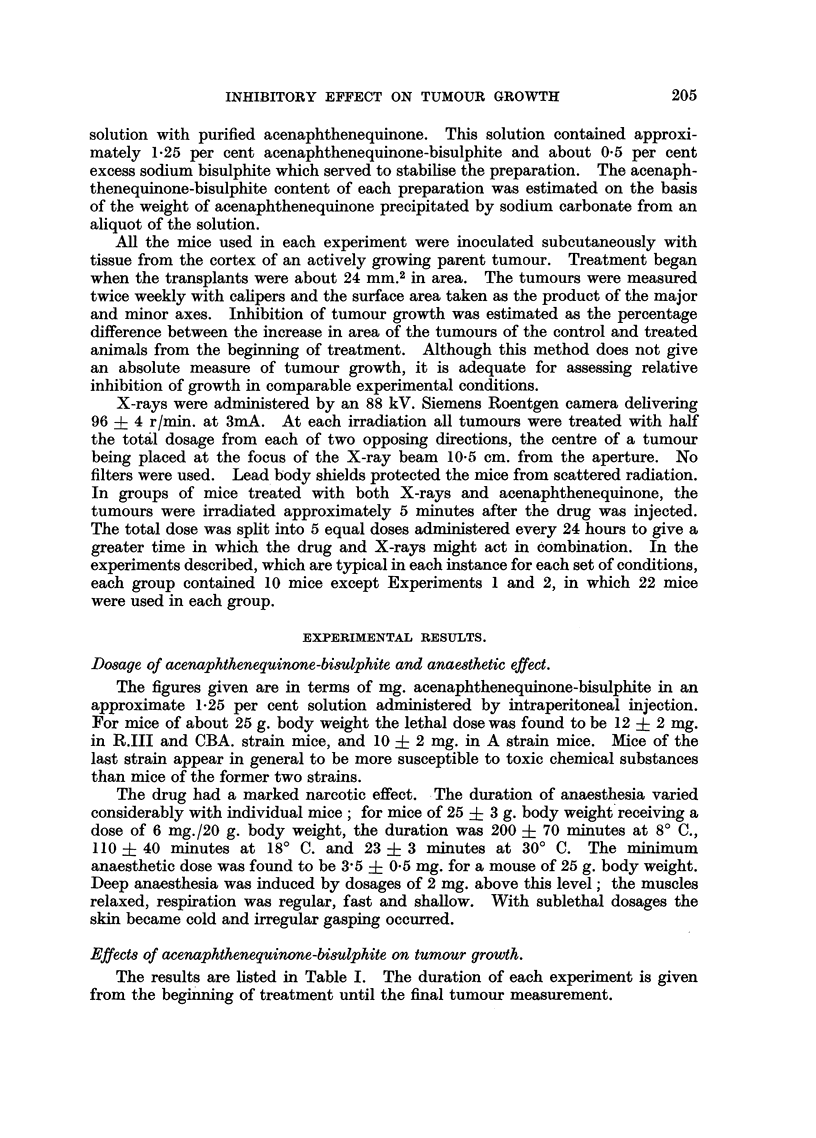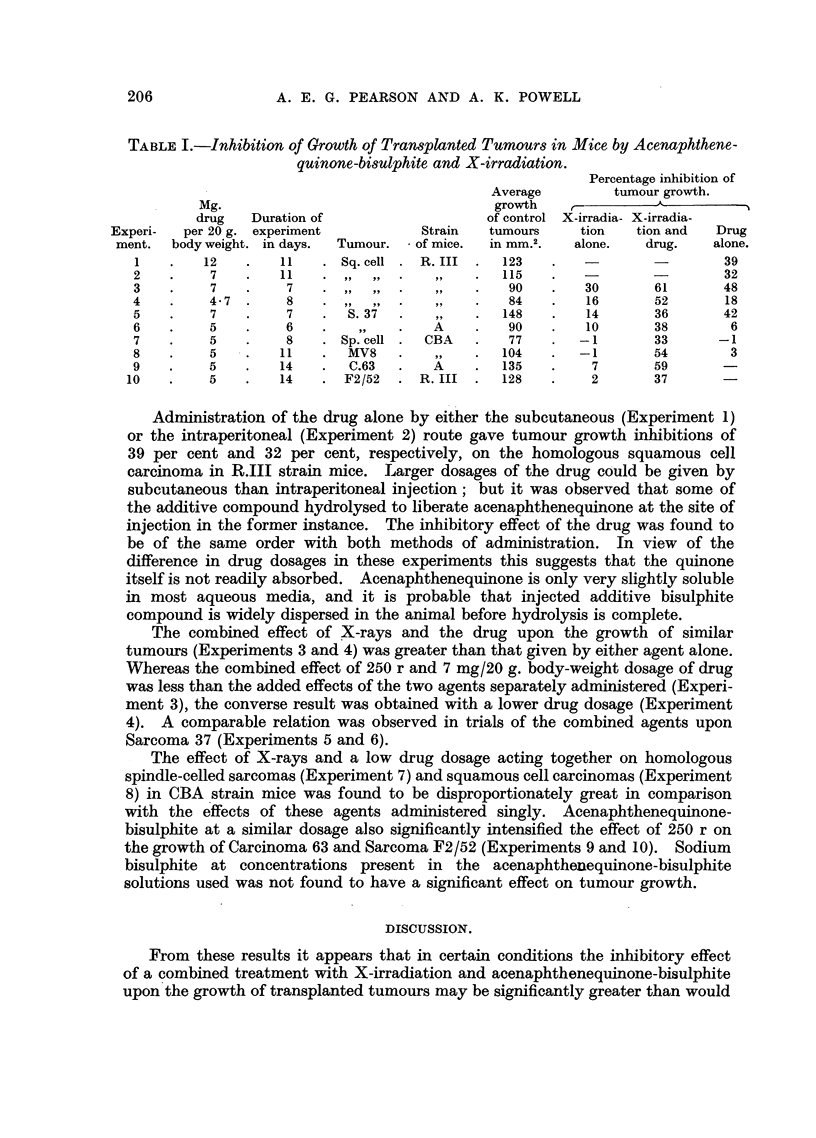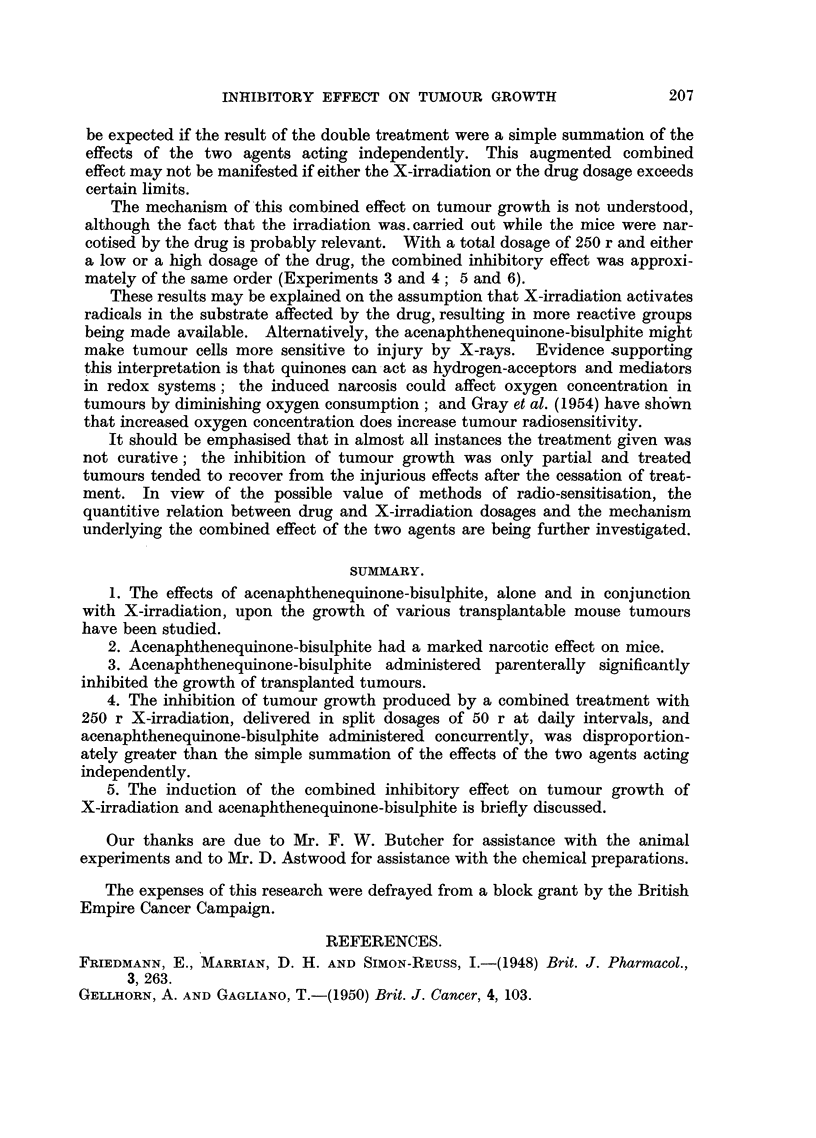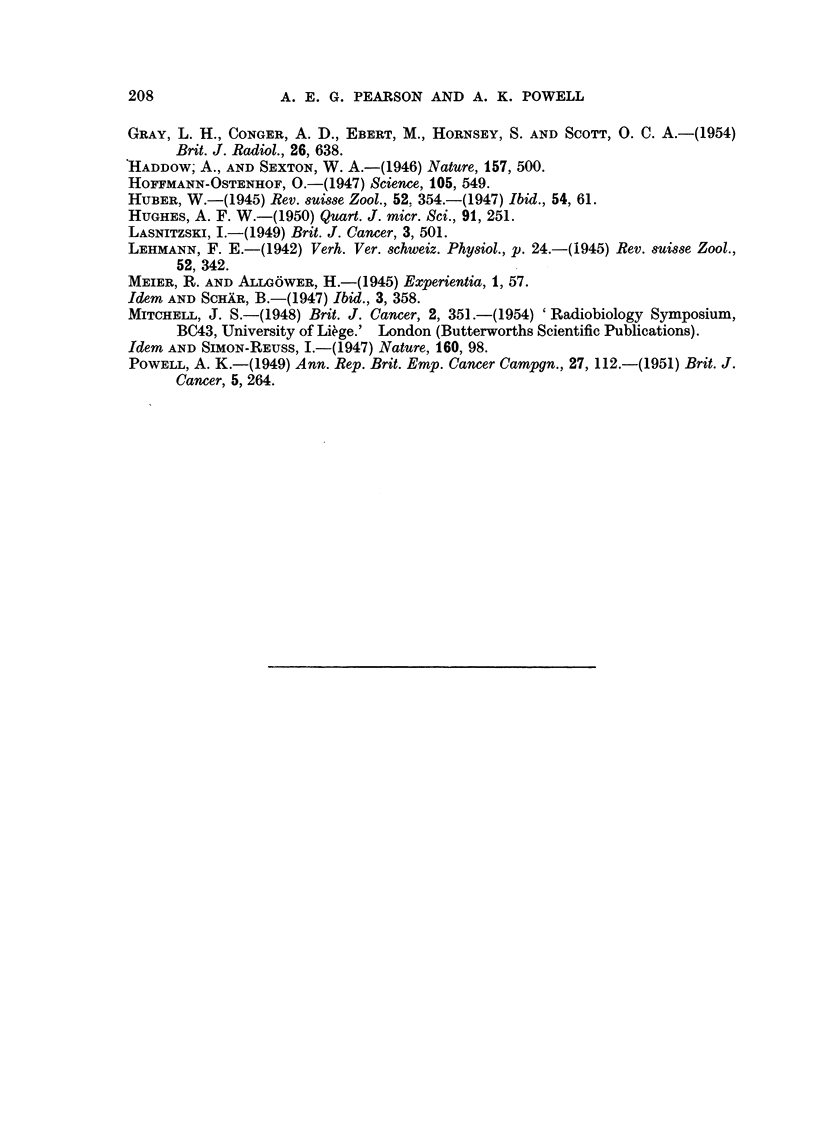# The Inhibitory Effect of Acenaphthenequinone-Bisulphite upon Tumour Growth in Mice

**DOI:** 10.1038/bjc.1955.15

**Published:** 1955-03

**Authors:** A. E. G. Pearson, A. K. Powell


### THE INHIBITORY EFFECT OF ACENAPHTHENEQUINONE-BISULPHITE UPON TUMOUR GROWTH IN MICE.

A. E. G. PEARSON AND A. K. POWELL.

*From the Department of Experimental Pathology, Mount Vernon Hospital, Northwood, Middlesex.*

Received for publication December 16, 1954.

QUINONES have been shown to be biologically active components by many workers. They have been reported to act as mitotic poisons on fibroblasts grown in tissue culture (Meier and Allgöwer, 1945 ; Meier and Schär, 1947) and on embryonic cells of the freshwater annelid *Tubifex* (Lehmann, 1942, 1945 ; Huber, 1945, 1947). Mitchell and Simon-Reuss (1947) described the inhibitory effect of tetrasodium 2-methyl-1 : 4-naphthohydroquinone (" Synkavit ") on normal and malignant cells grown *in vitro*. Friedmann, Marrian and Simon-Reuss (1948) have studied the antimitotic activity of 1 : 4-naphthohydroquinone diphosphate and its 2-methyl homologue on cultures of chick fibroblasts *in vitro*. The former was found to be 1000 times as active as the latter. Mitchell (1948) also reported increased radiosensitivity in tumours of patients concurrently treated with " Synkavit " and has recently (1954) reviewed later researches on this and related compounds. It was subsequently claimed by Gellhorn and Gagliano (1950) that " Synkavit " had no appreciable inhibitory effect on the growth of transplantable tumours in mice and rabbits. Quinones have considerable bacteriostatic powers (Hoffmann-Ostenhof, 1947). 9 : 10-Phenanthraquinone has a definite inhibitory effect on tumour growth in mice (Powell, 1951).

The narcotic urethane has been reported by Lasnitzski (1949) and Hughes (1950) to inhibit mitosis and by Haddow and Sexton (1946) to inhibit tumour growth in experimental animals. Acenaphthene-quinone, administered parenterally as the water-soluble sodium bisulphite additive compound, has a marked narcotic effect on experimental animals (Powell, 1949). This possession of narcotic properties by a quinonoid compound gave reason to investigate its effects upon tumour growth.

### MATERIALS AND METHODS.

The transplantable tumours used in this study were Carcinoma 63 in A strain mice, Sarcoma 37 in R.III and A strain mice, and the following homologous tumours : a spindle-celled sarcoma and a squamous cell carcinoma in CBA strain mice (MV8), a polymorphic sarcoma (F2/52) and a squamous cell carcinoma in R.III strain mice.

Acenaphthenequinone-bisulphite in aqueous solution was administered by intraperitoneal or subcutaneous injection. Injections were made once daily for either the duration of an experiment (Experiments 1, 2) or for five consecutive days (Experiments 3–10) when the mice were concurrently irradiated. The dosage of the drug was adjusted to the body weight of each mouse. The drug solution was prepared by saturating 1 per cent sodium metabisulphite in aqueous solution with purified acenaphthenequinone. This solution contained approximately 1·25 per cent acenaphthenequinone-bisulphite and about 0·5 per cent excess sodium bisulphite which served to stabilise the preparation. The acenaphthenequinone-bisulphite content of each preparation was estimated on the basis of the weight of acenaphthenequinone precipitated by sodium carbonate from an aliquot of the solution.

All the mice used in each experiment were inoculated subcutaneously with tissue from the cortex of an actively growing parent tumour. Treatment began when the transplants were about 24 mm.$^2$ in area. The tumours were measured twice weekly with calipers and the surface area taken as the product of the major and minor axes. Inhibition of tumour growth was estimated as the percentage difference between the increase in area of the tumours of the control and treated animals from the beginning of treatment. Although this method does not give an absolute measure of tumour growth, it is adequate for assessing relative inhibition of growth in comparable experimental conditions.

X-rays were administered by an 88 kV. Siemens Roentgen camera delivering 96 $\pm$ 4 r/min. at 3mA. At each irradiation all tumours were treated with half the total dosage from each of two opposing directions, the centre of a tumour being placed at the focus of the X-ray beam 10·5 cm. from the aperture. No filters were used. Lead body shields protected the mice from scattered radiation. In groups of mice treated with both X-rays and acenaphthenequinone, the tumours were irradiated approximately 5 minutes after the drug was injected. The total dose was split into 5 equal doses administered every 24 hours to give a greater time in which the drug and X-rays might act in combination. In the experiments described, which are typical in each instance for each set of conditions, each group contained 10 mice except Experiments 1 and 2, in which 22 mice were used in each group.

### EXPERIMENTAL RESULTS.

### *Dosage of acenaphthenequinone-bisulphite and anaesthetic effect.*

The figures given are in terms of mg. acenaphthenequinone-bisulphite in an approximate 1·25 per cent solution administered by intraperitoneal injection. For mice of about 25 g. body weight the lethal dose was found to be 12 $\pm$ 2 mg. in R.III and CBA. strain mice, and 10 $\pm$ 2 mg. in A strain mice. Mice of the last strain appear in general to be more susceptible to toxic chemical substances than mice of the former two strains.

The drug had a marked narcotic effect. The duration of anaesthesia varied considerably with individual mice ; for mice of 25 $\pm$ 3 g. body weight receiving a dose of 6 mg./20 g. body weight, the duration was 200 $\pm$ 70 minutes at 8° C., 110 $\pm$ 40 minutes at 18° C. and 23 $\pm$ 3 minutes at 30° C. The minimum anaesthetic dose was found to be 3·5 $\pm$ 0·5 mg. for a mouse of 25 g. body weight. Deep anaesthesia was induced by dosages of 2 mg. above this level ; the muscles relaxed, respiration was regular, fast and shallow. With sublethal dosages the skin became cold and irregular gasping occurred.

### *Effects of acenaphthenequinone-bisulphite on tumour growth.*

The results are listed in Table I. The duration of each experiment is given from the beginning of treatment until the final tumour measurement.

TABLE I.—*Inhibition of Growth of Transplanted Tumours in Mice by Acenaphthenequinone-bisulphite and X-irradiation.*

| Experiment. | Mg. drug per 20 g. body weight. | Duration of experiment in days. | Tumour. | Strain of mice. | Average growth of control tumours in mm.². | Percentage inhibition of tumour growth. X-irradiation alone. | X-irradiation and drug. | Drug alone. |
|---|---|---|---|---|---|---|---|---|
| 1 | 12 | 11 | Sq. cell | R. III | 123 | — | — | 39 |
| 2 | 7 | 11 | ,, ,, | ,, | 115 | — | — | 32 |
| 3 | 7 | 7 | ,, ,, | ,, | 90 | 30 | 61 | 48 |
| 4 | 4·7 | 8 | ,, ,, | ,, | 84 | 16 | 52 | 18 |
| 5 | 7 | 7 | S. 37 | ,, | 148 | 14 | 36 | 42 |
| 6 | 5 | 6 | ,, | A | 90 | 10 | 38 | 6 |
| 7 | 5 | 8 | Sp. cell | CBA | 77 | −1 | 33 | −1 |
| 8 | 5 | 11 | MV8 | ,, | 104 | −1 | 54 | 3 |
| 9 | 5 | 14 | C.63 | A | 135 | 7 | 59 | — |
| 10 | 5 | 14 | F2/52 | R. III | 128 | 2 | 37 | — |

Administration of the drug alone by either the subcutaneous (Experiment 1) or the intraperitoneal (Experiment 2) route gave tumour growth inhibitions of 39 per cent and 32 per cent, respectively, on the homologous squamous cell carcinoma in R.III strain mice. Larger dosages of the drug could be given by subcutaneous than intraperitoneal injection ; but it was observed that some of the additive compound hydrolysed to liberate acenaphthenequinone at the site of injection in the former instance. The inhibitory effect of the drug was found to be of the same order with both methods of administration. In view of the difference in drug dosages in these experiments this suggests that the quinone itself is not readily absorbed. Acenaphthenequinone is only very slightly soluble in most aqueous media, and it is probable that injected additive bisulphite compound is widely dispersed in the animal before hydrolysis is complete.

The combined effect of X-rays and the drug upon the growth of similar tumours (Experiments 3 and 4) was greater than that given by either agent alone. Whereas the combined effect of 250 r and 7 mg/20 g. body-weight dosage of drug was less than the added effects of the two agents separately administered (Experiment 3), the converse result was obtained with a lower drug dosage (Experiment 4). A comparable relation was observed in trials of the combined agents upon Sarcoma 37 (Experiments 5 and 6).

The effect of X-rays and a low drug dosage acting together on homologous spindle-celled sarcomas (Experiment 7) and squamous cell carcinomas (Experiment 8) in CBA strain mice was found to be disproportionately great in comparison with the effects of these agents administered singly. Acenaphthenequinone-bisulphite at a similar dosage also significantly intensified the effect of 250 r on the growth of Carcinoma 63 and Sarcoma F2/52 (Experiments 9 and 10). Sodium bisulphite at concentrations present in the acenaphthenequinone-bisulphite solutions used was not found to have a significant effect on tumour growth.

### DISCUSSION.

From these results it appears that in certain conditions the inhibitory effect of a combined treatment with X-irradiation and acenaphthenequinone-bisulphite upon the growth of transplanted tumours may be significantly greater than would be expected if the result of the double treatment were a simple summation of the effects of the two agents acting independently. This augmented combined effect may not be manifested if either the X-irradiation or the drug dosage exceeds certain limits.

The mechanism of this combined effect on tumour growth is not understood, although the fact that the irradiation was carried out while the mice were narcotised by the drug is probably relevant. With a total dosage of 250 r and either a low or a high dosage of the drug, the combined inhibitory effect was approximately of the same order (Experiments 3 and 4 ; 5 and 6).

These results may be explained on the assumption that X-irradiation activates radicals in the substrate affected by the drug, resulting in more reactive groups being made available. Alternatively, the acenaphthenequinone-bisulphite might make tumour cells more sensitive to injury by X-rays. Evidence supporting this interpretation is that quinones can act as hydrogen-acceptors and mediators in redox systems ; the induced narcosis could affect oxygen concentration in tumours by diminishing oxygen consumption ; and Gray *et al.* (1954) have shown that increased oxygen concentration does increase tumour radiosensitivity.

It should be emphasised that in almost all instances the treatment given was not curative ; the inhibition of tumour growth was only partial and treated tumours tended to recover from the injurious effects after the cessation of treatment. In view of the possible value of methods of radio-sensitisation, the quantitive relation between drug and X-irradiation dosages and the mechanism underlying the combined effect of the two agents are being further investigated.

### SUMMARY.

1. The effects of acenaphthenequinone-bisulphite, alone and in conjunction with X-irradiation, upon the growth of various transplantable mouse tumours have been studied.

2. Acenaphthenequinone-bisulphite had a marked narcotic effect on mice.

3. Acenaphthenequinone-bisulphite administered parenterally significantly inhibited the growth of transplanted tumours.

4. The inhibition of tumour growth produced by a combined treatment with 250 r X-irradiation, delivered in split dosages of 50 r at daily intervals, and acenaphthenequinone-bisulphite administered concurrently, was disproportionately greater than the simple summation of the effects of the two agents acting independently.

5. The induction of the combined inhibitory effect on tumour growth of X-irradiation and acenaphthenequinone-bisulphite is briefly discussed.

Our thanks are due to Mr. F. W. Butcher for assistance with the animal experiments and to Mr. D. Astwood for assistance with the chemical preparations.

The expenses of this research were defrayed from a block grant by the British Empire Cancer Campaign.

### REFERENCES.

FRIEDMANN, E., MARRIAN, D. H. AND SIMON-REUSS, I.—(1948) *Brit. J. Pharmacol.*, **3**, 263.

GELLHORN, A. AND GAGLIANO, T.—(1950) *Brit. J. Cancer*, **4**, 103.

Gray, L. H., Conger, A. D., Ebert, M., Hornsey, S. and Scott, O. C. A.—(1954) *Brit. J. Radiol.*, **26**, 638.
Haddow, A., and Sexton, W. A.—(1946) *Nature*, **157**, 500.
Hoffmann-Ostenhof, O.—(1947) *Science*, **105**, 549.
Huber, W.—(1945) *Rev. suisse Zool.*, **52**, 354.—(1947) *Ibid.*, **54**, 61.
Hughes, A. F. W.—(1950) *Quart. J. micr. Sci.*, **91**, 251.
Lasnitzski, I.—(1949) *Brit. J. Cancer*, **3**, 501.
Lehmann, F. E.—(1942) *Verh. Ver. schweiz. Physiol.*, *p.* 24.—(1945) *Rev. suisse Zool.*, **52**, 342.
Meier, R. and Allgöwer, H.—(1945) *Experientia*, **1**, 57.
*Idem* and Schär, B.—(1947) *Ibid.*, **3**, 358.
Mitchell, J. S.—(1948) *Brit. J. Cancer*, **2**, 351.—(1954) 'Radiobiology Symposium, BC43, University of Liège.' London (Butterworths Scientific Publications).
*Idem* and Simon-Reuss, I.—(1947) *Nature*, **160**, 98.
Powell, A. K.—(1949) *Ann. Rep. Brit. Emp. Cancer Campgn.*, **27**, 112.—(1951) *Brit. J. Cancer*, **5**, 264.